# Analysis of MnS Inclusions Formation in Resulphurised Steel via Modeling and Experiments

**DOI:** 10.3390/ma12122028

**Published:** 2019-06-24

**Authors:** Hui Liu, Delin Hu, Jianxun Fu

**Affiliations:** Center for Advanced Solidification Technology (CAST), School of Materials Science and Engineering, Shanghai University, Shanghai 200444, China; liuhui2015@shu.edu.cn (H.L.); amudesky@shu.edu.cn (D.H.)

**Keywords:** coupled model, MnS inclusion, solidification, segregation, growth

## Abstract

Controlling the formation of MnS inclusions during solidification influences the mechanical properties and machinability of the resulfurized steel. A coupled segregation–nucleation–growth model was developed by the finite-difference method involving solute redistribution, heterogeneous nucleation and growth kinetics. Laboratory solidification experiments were performed under various cooling rates in resulphurised 49MnVS steel. In this work, the influence of cooling rate on solute redistribution and growth size of MnS inclusions were simulated using the current coupled model, and the calculated results can provide a valuable reference for MnS formation. Increasing of the cooling rate led to early precipitation and refinement of formed MnS inclusions. Based on the simulation results and experimental data, mathematical relationships between the growing size of MnS with the cooling rate in the low ductility temperature region and in the whole solidification were obtained.

## 1. Introduction

Over the past few decades, MnS inclusions have been considered as detrimental species in steel matrix, and many effects have been made for desulfurization in the processes of hot metal pretreatment and converter steelmaking, etc [1]. However, the deep comprehension of MnS inclusions in the resulfurised steel matrix plays an essential role in the mechanical performance of steel products. MnS has a beneficial effect on the nucleation of intragranular ferrite and grain refinement [2,3,4]. Besides, wrapping the outside of MnS in harmful precipitates can improve the cutting performance and strength of steels [5]. However, the mechanical properties of steel exhibit anisotropy due to the good elongation of pure MnS inclusions, resulting in poor strength and ductility in steel plate [6,7,8]. Thus the comprehensive understanding and control of precipitation and growth of MnS inclusions during solidification are essential.

The evolution of MnS inclusions during cooling and solidification can be calculated and predicted using mathematical modeling. MnS is normally precipitated in the enriched remnant liquid during solidification and many models have been proposed to analyze the precipitation of MnS inclusions based on the redistribution of solute elements [9]. Clyne and Kurz [10] modified the back diffusion coefficient based on the model proposed by Brody and Flemings [11]. Subsequently, Ohnaka [12], Kobayashi [13], Hoseon [14], and Voller [15] developed models mainly focussing on the distribution of local solute concentration, and evaluated the precipitation of MnS. Further, the effects of multiple components, a columnar dendrite microstructure, and phase transformation were considered in a semi-empirical analytical model presented by Won and Thomas [16]. You et al. [17] proposed a model of MnS formation by taking into account the thermodynamics and kinetics in a simplified manner combined with Brownian motion collision. Meng et al. [18] simulated the precipitation of MnS using cellular automaton and demonstrated that the formation of MnS is accomplished by dendrite growth. A comprehensive model encompassing microsegregation, thermodynamics, and kinetics for describing and predicting the precipitation and growth of MnS needs further analysis.

In the present research, a new coupled model of MnS inclusions precipitation and growth during solidification was proposed based on previous work [19,20]. Using the current coupled model, the effect of cooling rate on the enrichment of solute elements and growth of MnS inclusions during solidification of a non-quenched and tempered 49MnVS steel were performed. In addition, laboratory experiments were carried out for validation of the model. 

## 2. Experimental 

**Figure 1:**
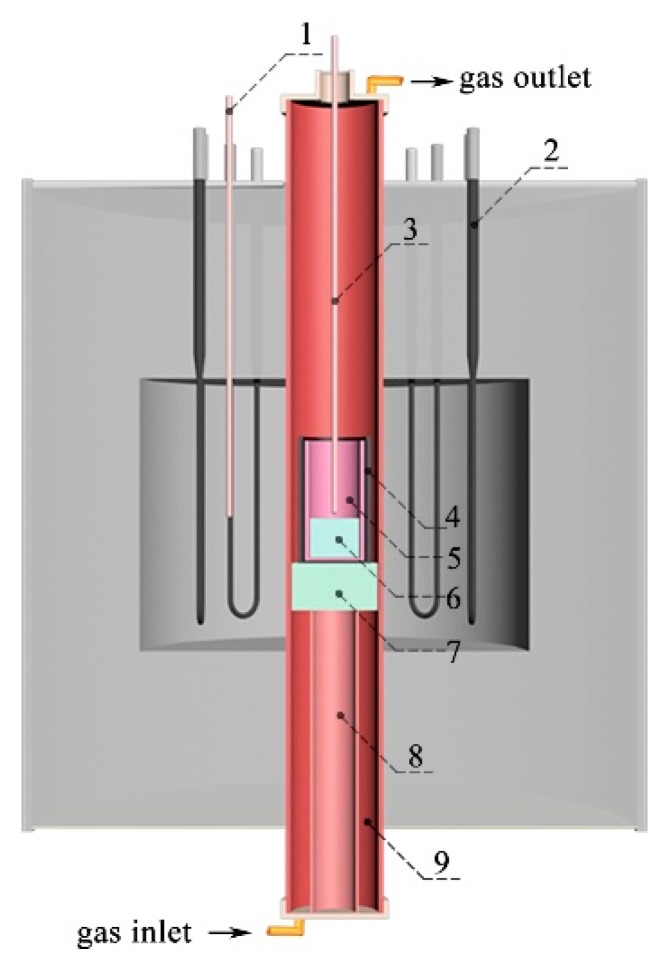
Schematic diagram of experimental equipment: **1** Thermocouple A; **2** Si Mo heating bars; **3** Thermocouple B; **4** graphite crucible; **5** Corundum crucible; **6** Specimen; **7** Heat insulation block; **8** Support tube; and **9** Corundum tube.

The experimental material used in the present study was a non-quenched and tempered resulphurised 49MnVS steel, and its main chemical compositions are shown in Table 1. The sample weighting 600 g was cut from a casting slab by an electric spark cutting machine. Afterward, the sample was ultrasonically cleaned in anhydrous ethanol for 5 min to ensure cleanliness. The prepared sample was set into a high purity alumina crucible (60 mm in diameter and 120 mm in height) which was then placed in the constant temperature zone of a tubular resistance furnace. The schematic diagram of the experimental equipment is shown in Figure 1. During the entire experiment process, High purity Ar gas was aerated into the reaction corundum tube with the flux of 1.00 L/min to prevent oxidation of the sample. The sample was heated up to 1550 °C for 30 min for full-homogenization and was then cooled to 1390 °C under different cooling rate, after which, it was quenched in water to room temperature. Finally, cylindrical ingots of different cooling rates (0.5, 1.0, 4.5, 8.0, and 15.0 °C/min, respectively) were obtained. Note that the actual cooling rate was determined using a thermocouple B, as seen in Figure 1.

After the experiment, a steel sample for metallographic observation was collected from the center of each ingot. Image-Pro Plus 6.0 software (manufacturer, Rockville, MD, USA) was used to measure the average length of diameters at 2-degree intervals and passing through the object’s centroid. The steel sample was polished and cleaned in ethanol for 5 min using a ultrasonic cleaner (Buehler, Lake Bluff, IL, USA). Besides, to obtain the tri-dimensional morphologies of MnS, the non-aqueous electrolytic method presented by Janis et al [21] was adopted to avoided a serious dissolution of MnS. 10%AA electrolyte (10 vol.% acetylacetone-1 vol.% tetramethylammonium chloride-methanol) was employed in the present study [22]. The processed sample was characterized by an optical microscope (Carl Zeiss, A2m, Jena, Germany) and a scanning electron microscope combined with Energy Dispersive Spectrometer (SEM-EDS) (Phenom, Pro X, Eindhoven, Netherlands).

## 3. Coupled Model of Solute Segregation and Inclusion Growth 

**Figure 2:**
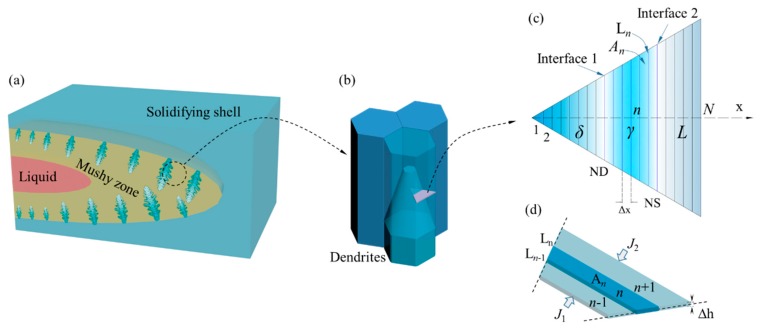
Schematic diagram of the micro-segregation model for continuous casting: (**a**) dendritic growth in the mushy zone; (**b**) dendrite array morphology; (**c**) calculation domain; and (**d**) diffusion calculation in single phase internal nodes.

Figure 2 presents a solidification process during the continuous casting, where dendrites grow from the solidifying shell in the mushy zone. The transverse cross section of dendrites is approximated by a regular hexagon, and the formed dendrites are closely arranged, as shown in Figure 2b. The concentration of solute elements in liquid is assumed to be uniform along the direction of dendrite growth [23], and the elements diffusion in the direction of dendrite axis is negligible. Consequently, the one-dimensional diffusion was simplified from the three-dimensional diffusion in dendrite growth, as shown in Figure 2c. From our previous studies [20], the optimized equation was established describing the diffusion rate of solid-liquid phases, as shown in Equation (1).

(1) $$C_{n(\Delta t)}^{\prime }=D\frac{(C_{n-1(\Delta t)}-C_{n(\Delta t)})L_{n-1}+(C_{n+1(\Delta t)}-C_{n(\Delta t)})L_{n}}{\Delta xA_{n}}$$

where $C_{n(\Delta t)}^{\prime }$ is real-time concentration in a time step (wt.%), *D* is the diffusion coefficient (cm^2^/s), Δ*t* and Δ*x* are the time step and the space step, *L*_n_ is the length of the interface between node n and node n + 1 (m), while *A*_n_ is the area of node n (m^2^). In addition, MnS preferentially precipitates in the interdendritic area when the product of Mn and S concentrations exceeds the equilibrium value in remnant liquid [19]. The distribution of temperature is determined by cooling rate and assumed to be uniform. The δ-phase develops from the liquid phase with the procedure of solidification. The γ-phase generates in two ways, one is from the interface between δ-phase and liquid phase and the other from the last-part solidification under the condition that the δ/γ transformation occurs after complete solidification. Additionally, the solute elements are equilibrium distributed at both solid/liquid interface and the δ/γ interface. It is worth mentioning that the above assumptions are reasonable in a wide range of casting process.

### 3.1. Thermodynamics of MnS Precipitation Process

The formation reaction of MnS in steel is expressed as [Mn] + [S] = (MnS), and the equilibrium constant of the reaction is shown in Equation (2). MnS begins to precipitate in the remnant liquid when the supersaturation of Mn and S occurs.

(2) KMnS=aMnSa[Mn]⋅a[S]=aMnSfMnT[%Mn]fST[%S]=10exp(8627T−4.745)

where *K*_MnS_ is the equilibrium constant of the reaction [Mn] + [S] = (MnS), *T* is the temperature in K. *a*_MnS_ = 1 is the activity of pure MnS, $f_{Mn}^{T}$ and $f_{S}^{T}$ are the henry activity coefficients and were determined as follows:

(3) logfiT=(2538T−0.355)logfi1873K

(4) $$\log f_{i}^{1873K}=e_{i}^{j}\left[\%i\right]+\sum e_{i}^{j}\left[\%j\right]$$

where $f_{i}^{1873K}$ is the activity coefficient of the solute element in the liquid phase at 1873 K and were calculated according to the Wagner’s formula [24], [%*i*] and [%*j*] are the concentrations (wt.%) of solute elements *i* and *j*, and $e_{i}^{j}$ is the interaction coefficient of element *j* to *i*; as listed in Table 2.

According to the equilibrium relationship of the above formula, it can be concluded that the consumption of solute elements in the liquid phase.

(5) $$(C_{L,\mathrm{Mn}}-\Delta C_{L,\mathrm{Mn}})\cdot f_{\mathrm{Mn}}^{T}\cdot (C_{L,S}-\Delta C_{L,S})\cdot f_{S}^{T}\cdot K_{\mathrm{MnS}}=1$$

where $C_{L,Mn}$ and $C_{L,S}$ are the local concentrations of Mn and S in the residual liquid phase, respectively, $\Delta C_{L,Mn}$ and $\Delta C_{L,S}$ are the solute element consumption of Mn and S caused by the formation of MnS.

Assuming that all the consumption of Mn and S are used to generate MnS, the precipitation amount of MnS can be calculated through the above equations as expressed in the following equation:

(6) $$w_{\mathrm{MnS}}=\Delta C_{L,\mathrm{Mn}}+\Delta C_{L,S}=\frac{b+1}{2b}(C_{L,\mathrm{Mn}}+bC_{L,S}-\sqrt{{\left(C_{L,\mathrm{Mn}}+bC_{L,S}\right)}^{2}-4b(C_{L,\mathrm{Mn}}C_{L,S}-\frac{1}{K_{\mathrm{MnS}}^{}\cdot f_{\mathrm{Mn}}^{}\cdot f_{S}^{}})}$$

where *w*_MnS_ is the precipitation amount of MnS, and *b* is the ratio of $\Delta C_{L,Mn}$ and $\Delta C_{L,S}$, and its value is equal to the ratio of *M*_Mn_ (the molar mass of Mn) to *M*_S_ (the molar mass of S) resulting from the equilibrium consumption of solute Mn and S.

### 3.2. Nucleation 

It has been often reported that the nucleation of MnS preferentially occurs at grain boundaries and dislocations [25,26]. The nucleation of MnS was based on the classical homogeneous nucleation process, and the relationship between the critical free energy of heterogeneous nucleation ($\Delta G_{\mathrm{he}}^{*}$) and homogeneous nucleation ($\Delta G_{\mathrm{ho}}^{*}$) are presented through

(7) $$\Delta G_{\mathrm{he}}^{*}=\frac{1}{2}\left(2-3\cos\theta +\cos^{3}\theta \right)\Delta G_{\mathrm{ho}}^{*}$$

and

(8) $$\cos\theta =0.5\sigma_{B}/\sigma$$

where *σ*_B_ and *σ* are the surface energy of matrix and precipitates (J/m^2^), and *θ* is the contact angle.

It is reasonable to neglect the influence of elastic energy because it can be completely released during the solidification process. Assuming that the precipitated MnS nuclei is a sphere of radius, the critical free energy of homogeneous nucleation is given by Equation (9).

(9) $$\Delta G_{\mathrm{ho}}^{*}=\frac{4}{3}\pi r_{0}{}^{3}\Delta G_{V}+4\pi r_{0}{}^{2}\sigma =\frac{16}{3}\pi \frac{\sigma^{3}}{\Delta G_{V}{}^{2}}$$

where *r*_0_ is the critical radius of MnS, and Δ*G*_V_ is the free energy per unit volume. The chemical driving force associated with the unit volume of MnS can be represented by Equation (10) [27].

(10) $$\Delta G_{V}=\frac{-\left(\Delta G^{\theta }+RT\ln J_{P}\right)}{V_{\mathrm{MnS}}}=-\frac{RT}{V_{\mathrm{MnS}}}\ln\frac{a_{\mathrm{Mn}}^{ac}\cdot a_{S}^{ac}}{a_{\mathrm{Mn}}^{eq}\cdot a_{S}^{eq}}$$

where *V*_MnS_ is the molar volume of MnS (22.3 L/mol), $a_{Mn}^{ac}$ and $a_{S}^{ac}$ are the actual activities of elements Mn and S, $a_{Mn}^{eq}$ and $a_{S}^{eq}$ are the equilibrium activities of elements Mn and S. 

The nucleation rate is calculated based on a classical nucleation law as expressed in the following equations [28,29].

(11) $${\frac{dN}{dt}|}_{\mathrm{Nucleation}}=N_{0}Z\beta^{\prime }\exp\left(-\frac{\Delta G^{*}}{k_{b}T}\right)\exp\left(-\frac{\tau }{t}\right)$$

(12) $$N_{0}=0.5\rho^{1.5}$$

(13) $$\beta^{\prime }=4\pi r_{0}{}^{2}D_{i}^{d}\cdot C_{i}^{d}/a_{0}{}^{4}$$

whereas, *N*_0_ is the number of nodes in the dislocation network, *Z* is the Zeldovich constant (0.05), *β*’ is the atomic impingement rate, *k_b_* is the Boltzmann constant (1.38065 × 10^−23^ J/K), τ is the incubation time, the precipitation of MnS on dislocations is assumed to be instantaneous and therefore, the incubation time is considered to be zero, *t* is growth time, *ρ* is the dislocation density, which is about 10^13^ m^−2^ in low carbon steel, $C_{i}^{d}$ and $D_{i}^{d}$ represent the concentration and diffusion coefficient of the rate determining elements, in the MnS formation system the determining species is Mn or S, and *a*_0_ is the lattice parameter. Moreover, the main kinetic parameters in formation of MnS are shown in Table 3 [30]. 

### 3.3. Growth of Nuclei 

**Figure 3:**
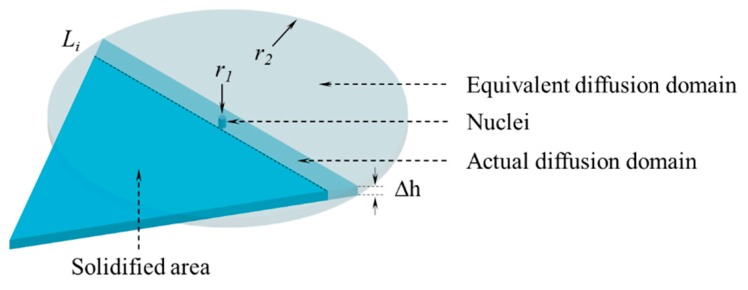
Schematic diagram of MnS precipitation in the calculation domain.

A schematic diagram of MnS precipitation in the calculation domain is presented in Figure 3. The product of Mn and S concentrations exceeds the equilibrium value during crystallization and hence, MnS precipitates in the remnant liquid. As mentioned earlier, the triangle area is the calculation domain. The remnant liquid area where MnS begins to precipitate is referred to as the actual diffusion domain of determining element. Elements diffusion domain in the remnant liquid is asymmetric, but the local diffusion process is random and symmetrical in space. The circular symmetric region is reasonably considered as the equivalent diffusion domain for MnS formation, and the relationship between the equivalent diffusion domain and the actual diffusion domain can be corrected by a conversion factor (*k*_ae_), as expressed in Equation (14). Thus MnS with radius *r*_1_ is considered to grow in a dynamic circular diffusion domain with radius *r*_2_.

*k*_ae_ = *A*_a_/*A*_e_ (14)

where *A*_e_ and *A*_a_ are the areas of equivalent diffusion domain and actual diffusion domain.

The rates of diffusion into solid and liquid phases are calculated according to the Fick’s second law, as expressed in Equation (15).

(15) $$\frac{\partial C}{\partial t}=\frac{1}{r}\left[\frac{\partial }{\partial r}\left(rD\frac{\partial C}{\partial r}\right)+\frac{\partial }{\partial \theta }\left(\frac{D}{r}\frac{\partial C}{\partial \theta }\right)+\frac{\partial }{\partial z}\left(rD\frac{\partial C}{\partial z}\right)\right]$$

where *r*, *θ*, and *z* are three coordinate variables in the column coordinate system, based on assumptions as described before, the diffusion of solute elements is locally steady on the cylindrical surface and therefore, the diffusion coefficient does not vary with the solute concentration. The diffusion rate of solid–liquid phases can be simplified as shown in Equation (16), while diffusion flux on different cylindrical surface is shown in Equation (17).

(16) $$\frac{\partial C}{\partial t}=\frac{D}{r}\left[\frac{\partial }{\partial r}\left(r\frac{\partial C}{\partial r}\right)\right]$$

(17) $$J=\frac{dm}{Adt}=-D\frac{dC}{dr}$$

As shown in Figure 3, a stable MnS nuclei is formed from the residual liquid. In symmetric steady-state diffusion, the initial conditions and boundary conditions of concentration distribution are *C* = *C*_1_ at *r* = *r*_1_ and *C* = *C*_2_ at *r* = *r*_2_, respectively. Therefore, the distribution of concentration can be calculated using the above boundary conditions, as shown in Equation (18).

(18) $$C\left(r\right)=\frac{C_{1}-C_{2}}{\ln r_{1}-\ln r_{2}}\ln r+\frac{C_{2}\cdot \ln r_{1}-C_{1}\cdot \ln r_{2}}{\ln r_{1}-\ln r_{2}}$$

where *J* is the diffusion flux, and *A* is the surface area of formed MnS in the cylindrical coordinate system. The mass balance surrounding one MnS nuclei is expressed in Equation (19).

(19) $$2\pi r\Delta hJ\Delta t=\rho_{\mathrm{MnS}}\pi \left[{\left(r+\Delta r\right)}^{2}-r^{2}\right]$$

Combining Equations (16–19), the differential form of MnS growth is obtained as expressed in Equation (20).

(20) $$\frac{dr}{dt}=\frac{1}{r}\frac{D}{\rho_{\mathrm{MnS}}}\frac{C_{1}-C_{2}}{\ln r_{1}-\ln r_{2}}$$

As evident in the above equation, the direct driving force for MnS growth is the local concentration gradient of the determining elements rather than concentration difference.

### 3.4. Calculation Solution of the Model 

The precipitation and growth of interdendritic MnS were mainly studied in the present coupled model, and the secondary dendrite arm spacing is calculated using Equation (21) which was proposed by Won et al. [16].

(21) $$\lambda_{2}=\left\{ \begin{array}{l} (169.1-720.9\cdot w_{C})\cdot R_{C}{}^{-0.4935},\;0<w_{C}\leq 0.15\\ 143.9\cdot R_{C}{}^{-0.3616}\cdot w_{C}{}^{\left(0.5501-1.996\cdot C_{C}\right)},\;0.15<w_{C} \end{array}\right.$$

where *λ*_2_ is secondary dendrite arm spacing (µm), *w*_C_ is the carbon content (wt.%) and *R*_C_ is the cooling rate (°C/s).

**Figure 4:**
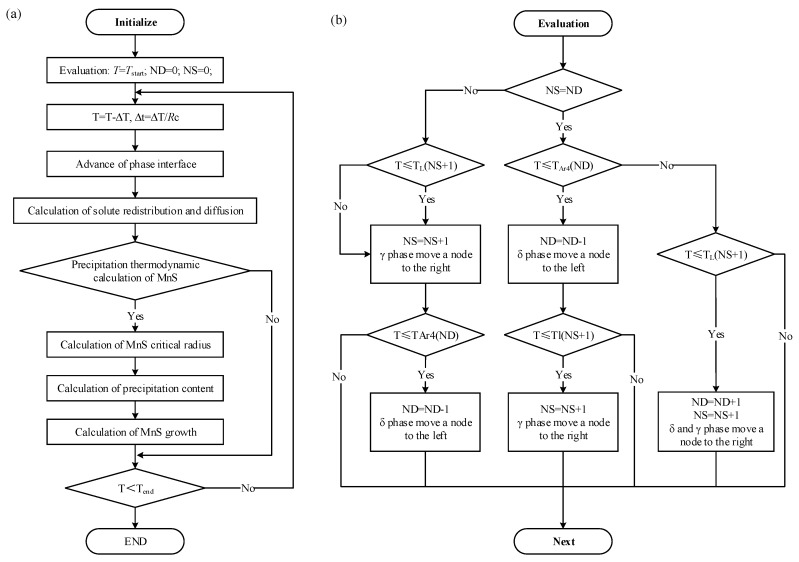
The flow chart: (**a**) the coupled segregation–nucleation–growth model; (**b**) phase interface advancing and judgment.

A flow chart of the present coupled model for the formation of MnS is displayed in Figure 4. The input parameters for the model are starting temperature (*T*_start_), cooling rate (*R*_C_), and initial composition of the steel. The phase transverse are considered and the advancing of phase interface and the algorithm is shown in Figure 4b. The determination of the solid/liquid interface movement at each temperature step is as described below. 

When the carbon content in steels exceeds 0.53%, the first crystallized phase at the center of dendrite is γ-phase. Otherwise, δ-phase is first generated in the steel liquid during solidification. The δ/γ transformation temperature (*T*_ar4_) is calculated using the solute concentrations in solid phase at the solid /liquid interface. When the liquidus temperature (*T*_L_) and the δ/γ transformation temperature (*T*_ar4_) of one node become equal to the actual temperature, the solidification and δ/γ transformation in the node are considered to be complete, and the interface is moved to the next node. The *T*_L_ and *T*_ar4_ are calculated using Equations (22) and (23) [16,31].

(22) $$T_{L}=T_{\mathrm{Fe}}-\sum m_{i}\cdot C_{L,i}$$

(23) $$T_{\mathrm{Ar}4}=T_{\mathrm{Fe}}^{\delta /\gamma }-\sum n_{i}\cdot C_{\delta ,i}$$

where *T*_Fe_ and $T_{\mathrm{Fe}}^{\delta /\gamma }$ are the melting temperature of pure iron (1536 °C) and the δ/γ transformation temperature of pure iron (1392 °C), *m*_i_ and *n*_i_ are the slopes of liquidus and Ar4 line according to the pseudobinary Fe-phase diagram, *C*_L,i_ and *C*_δ,i_ are the solute concentration of element *i* in liquid and solid phase (wt.%).

In addition, considering the diffusion of solute elements in the solid phase, the relationship between the diffusion coefficient and temperature can be calculated according to the Arrhenius formula as shown in Equation (24).

(24) Di=D0exp(−QRT)

where *D*_0_ is diffusion constant (cm^2^/s), *Q* is the activation energy (J/mol), *R* is the gas constant (8.314 J/(kg⋅K)), *T* is temperature (K). The equilibrium partition coefficients and diffusion coefficient of the solute elements are list in Table 4 [32,33].

With the temperature decreasing and solid /liquid interface advancing, the thermodynamics and kinetics of MnS formation were calculated by step. During the solidification, the formation of new phase causes the redistribution of solute in the remnant liquid. MnS precipitates in the remnant liquid when the product of Mn and S concentrations exceeds the equilibrium value. In the meantime, stable MnS nuclei is generated when the nucleation radius of MnS exceeds the critical value, otherwise the unstable MnS dissolves in the liquid. The precipitation of MnS was continuously simulated in combination with the enrichment of solute elements. Accordingly, the subsequent calculation started with the new temperature or time step and terminated at the end-temperature. 

The material used in this simulation is resulphurised 49MnVS steel, whose chemical composition is shown in Table 1. The calculation procedure was done at 30 °C higher than the liquidus temperature of the steel, and the influence of cooling rate on solute segregation and formation of MnS inclusions are analyzed in the current work.

## 4. Analysis and Discussion

### 4.1. Effect of Cooling Rate on MnS Formation 

**Figure 5:**
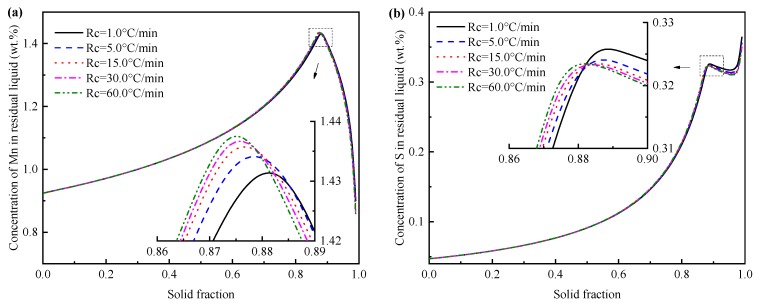
Effect of cooling rate on (**a**) Mn and (**b**) S content in the residual liquid.

The concentration of Mn and S in residual liquid under various cooling conditions are shown in Figure 5. The concentration of Mn and S increase gradually with the solidification process, the enrichment of S is greater than Mn. MnS begins to precipitate after the reduction of Mn and S content. Segregation ratio indicates the enrichment of solute concentration and can be expressed using the ratio of local concentration to the initial value. As evidenced in Figure 5, the segregation ratio of Mn and S are approximately 1.57 and 6.87 at the beginning of MnS formation. The amount of Mn consumption for sulfides precipitation is larger than the amount of Mn rejected from the solidifying metal, thus the rapidly decreasing of Mn content occurs. Besides, the enrichment of S first decreases and then increases due to the low solubility of S in solid. The increasing cooling rate leads to the advance of the MnS precipitation, and the precipitation solid fraction (*f*_S_) of MnS are 0.874, 0.875, 0.876, 0.878 and 0.881 at the cooling rate of 1.0, 5.0, 15.0, 30.0 and 60.0 °C/min, respectively. 

**Figure 6:**
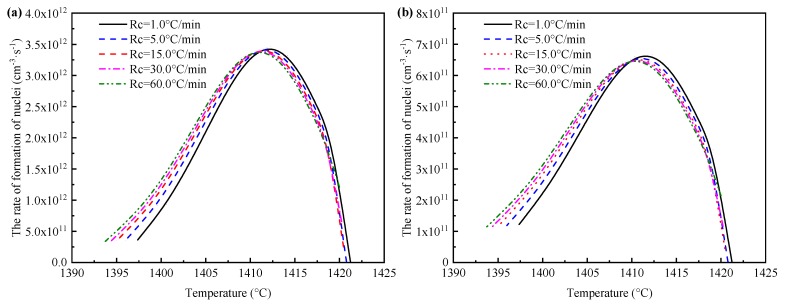
Effect of cooling rate on the nucleation rate of MnS: (**a**) determining element is Mn; (**b**) determining element is S.

The solute element of Mn and S were considered as the determining species in the current calculation, respectively. The effect of cooling rate on the nucleation rate of MnS is presented in Figure 6. The rate of formation of MnS nuclei first increase and then decrease with solidification, and the formation rate of MnS nuclei shows the same trend at various cooling rates. It is found that when Mn is the limiting element, the nucleation rate of MnS is one order of magnitude higher than that when S is the limiting element. Therefore, it can be concluded that element S is the determining species in MnS precipitation. However, due to the high partition coefficient of element Mn and its continuous consumption during the growth process of MnS, the concentration of solute Mn plays a restrictive role in determining the precipitated amount of MnS.

**Figure 7:**
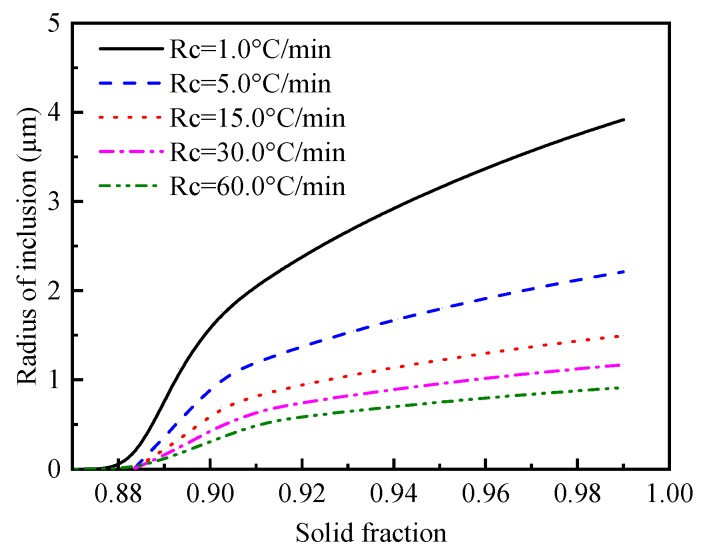
Effect of cooling rate on the size of MnS particles.

The variations of the radius of MnS particles during solidification for the five cooling rates is illustrated in Figure 7. The results show that the growth rate of MnS increases rapidly first and then slows down with time. At the end of the solidification corresponding to a solid fraction of 0.99, the diameters of the MnS particles are about 7.83, 4.42, 2.99, 2.34 and 1.83 μm under the cooling rate of 1.0, 5.0, 15.0, 30.0 and 60.0 °C/min, respectively. The radius of MnS particles reduces with the increase of cooling rate. During the precipitation and growth period of MnS particle, the local growth time increases with the decreasing cooling rate. It can be concluded that the MnS diameter changes with the cooling rate as a result of the enrichment and local diffusion time of solute elements.

### 4.2. Observation of Samples and Analysis of Inclusions

**Figure 8:**
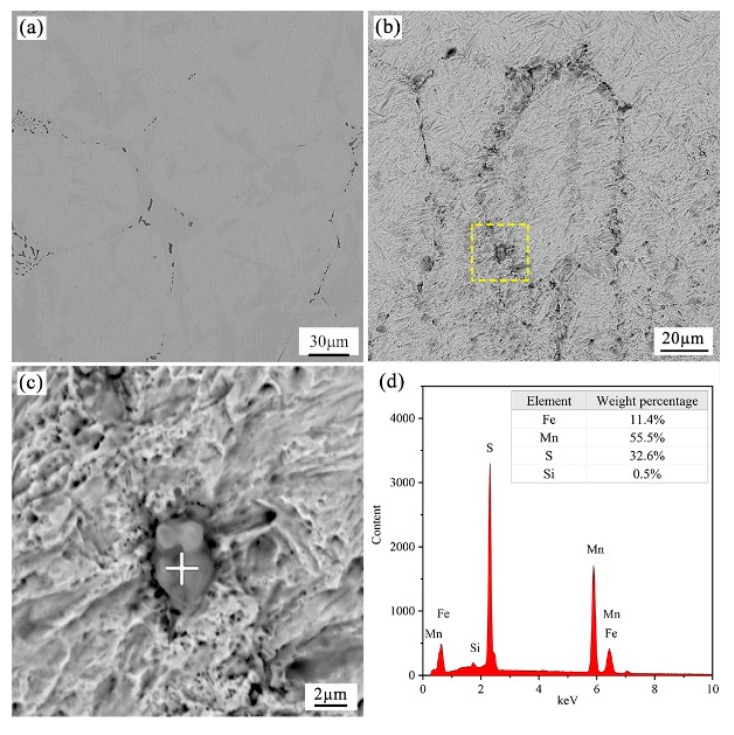
The morphologies and EDS analysis of sample inclusions under cooling rate of 1.0 °C/min: (**a**) the typical two-dimensional morphologies of inclusions; (**b**) the typical tri-dimensional morphologies of inclusions; (**c**) magnification of inclusion; and (**d**) EDS analysis of the inclusion.

**Figure 9:**
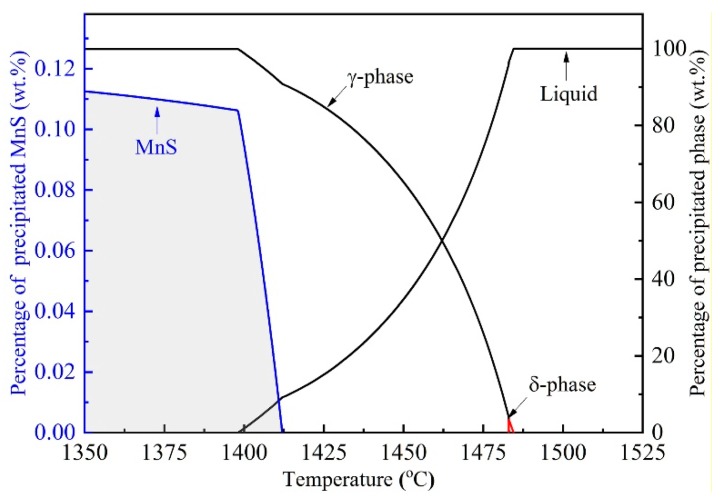
MnS precipitation and phase transformation during solidification of 49MnVS steel.

Figure 8 shows the typical two- and tri-dimensional morphologies of inclusions in a sample under the cooling rate of 1.0 °C/min, the results reveal that almost all the inclusions present a unified dark gray in the view of backscatter mode of the SEM. Energy spectrum analysis presented in Figure 8d indicates that the formed inclusions in rod-like or spindle shape mainly comprised of MnS. The distribution of MnS inclusions present a similar trend in various cooling samples, and most of the MnS precipitate along the grain boundaries. In addition, the equilibrium solidification of 49MnVS steel was calculated using FactSage 7.2 software (ThermFact Ltd., Montreal, Canada and GTT-Technologies, Aachen, Germany) with the FactPS, Ftoxid, and FSstel databases [34,35,36], as presented in Figure 9. MnS begins to precipitate from 1411.0 °C at which the liquid, γ phase and MnS co-exist. Subsequently, MnS rapidly precipitates in the mushy zone during the late stage of solidification. The formation of MnS is accomplished with the crystallization of γ phase, and therefore it is reasonable that the observed precipitated MnS shows chain-like patterns along the grain boundaries in the present cooling sample. 

**Figure 10:**
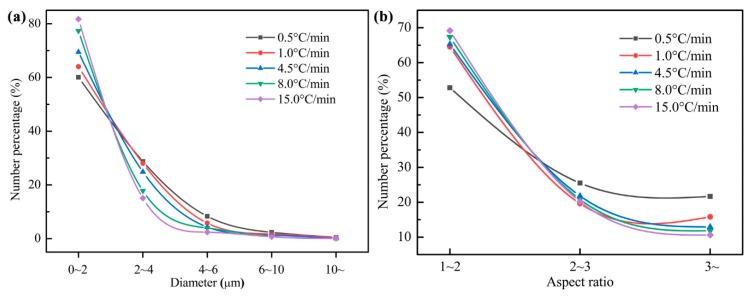
Influence of the cooling rate on (**a**) the size distribution of MnS and (**b**) aspect ratio of MnS.

The size distribution of MnS inclusion under various cooling conditions is shown in Figure 10a, which shows that most of the MnS inclusions in the present steel sample are within 4 μm. It is noteworthy that the quenching temperature adopted in the present study was 1390 °C, the growth time for MnS formation is not sufficient and the in-situ state of the inclusions was preserved under different cooling conditions. As presented in Figure 10a, it is found that the size of the MnS inclusions increases with the increasing cooling rate in the smallest class size (0~2 μm), and the opposite trend is obtained in the other classes. The influence of cooling rate on the aspect ratio (*A*_R_) of MnS is shown in Figure 10b, most of the MnS inclusions in the sample are spherical (*A*_R_ ≤ 2), followed by ellipsoidal (2 < *A*_R_ ≤ 3) and rod-like (*A*_R_ > 3). The percentage of spherical MnS inclusions increases with increasing cooling rate, and the percentage of ellipsoidal and rod-like MnS inclusions decrease with increasing cooling rate. According to the above discussion, it is concluded that for studied 49MnVS steel, increasing cooling rate is beneficial to the formation of ellipsoidal and tiny MnS inclusions.

**Figure 11:**
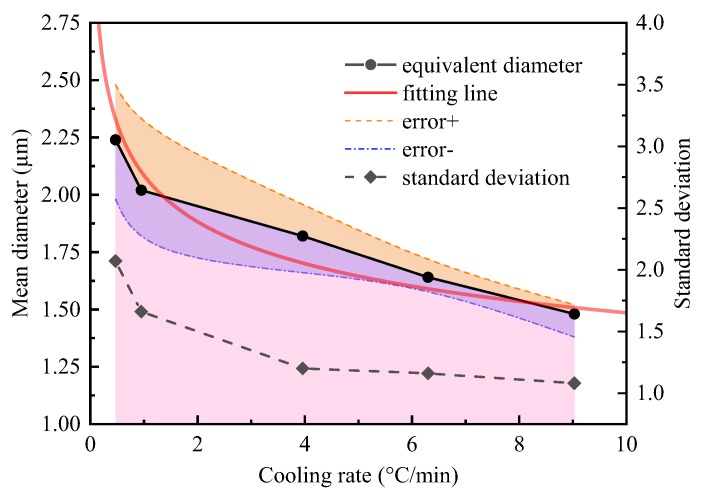
Influence of actual cooling rate on the mean diameter of MnS inclusion.

The actual cooling rate (measured by thermocouple B) of each sample is 0.47, 0.95, 3.96, 6.30 and 9.03 °C/min corresponding to the setting cooling rate (measured by thermocouple A) of 0.5, 1.0, 4.5, 8.0 and 15.0 °C/min, respectively. The mean diameter of MnS inclusion (*d*_MnS_) in various cooling samples are shown in Figure 11, which shows that the *d*_MnS_ decreases with increasing of the actual cooling rate (*υ*). The value of *d*_MnS_ is 2.24, 2.02, 1.82, 1.64 and 1.48 μm at actual cooling rate of 0.47, 0.95, 3.96, 6.30 and 9.03 °C/min, respectively. While the standard deviation of *d*_MnS_ is 2.07, 1.66, 1.20, 1.16 and 1.08. 

To detect the correlations between *d*_MnS_ and *υ*, a minimum square method was used for regression analysis based on the experimental data. A mathematical expression was given in Equation (25), and the regression coefficients (*R*^2^) was 0.954. In addition, the fitting curve was present in Figure 10, and the proximity of the fitting curve to the data points reflected the accuracy of regressive relationship.

(25) $$d_{\mathrm{MnS}}=2.040\upsilon^{-0.123}$$

### 4.3. Comparison Between Experiment and Calculation

Based on the coupled model, the simulated diameter of MnS was 7.83, 4.42, 2.99, 2.34 and 1.83 μm at the cooling rate of 1.0, 5.0, 15.0, 30.0 and 60.0 °C/min. Similarly, the relationship between cooling rate and calculated MnS diameter was obtained via linear regression analysis as shown in Equation (26), and *R*^2^ was 0.999.

(26) $$d_{\mathrm{MnS}}=7.830\upsilon^{-0.355}$$

**Figure 12:**
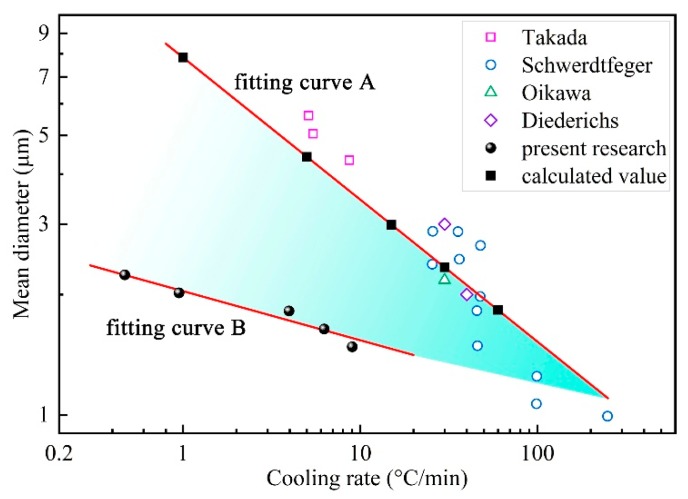
Comparison of the effect of cooling rate on MnS size.

The comparison of experimental data and calculated value versus cooling rate in common logarithmic coordinates are shown in Figure 12. The growth of MnS in the low ductility temperature region and the whole solidification process are described by the fitting curve A and B, as expressed in Equations (25) and (26). To evaluate the accuracy of the present coupled model, the experimental data carried out by Schwerdtfeger [37], Takada [38], Oikawa [39], and Diederichs [40] were used for verification. The materials used in the previous papers [37,38,39,40] were low and medium carbon steel, and the sulfur content are similar to that of the steel in the current study. As presented in Figure 12, the proximity of the previous research data points to the fitting curve A reflecting the accuracy of the regressive relationship. It can be concluded that Equation (26) seems to adequately characterize the variations of MnS diameter with the cooling rate. 

It should be pointed out that the present sample was obtained at a quenching temperature of 1390 °C. The solidus temperature of studied 49MnVS steel is 1391 °C according to the equilibrium calculation of MnS precipitation, as shown in Figure 9. However, owing to the enrichment of solute elements, the actual solidus temperature is lower than the calculated value, which means solid–liquid coexistence when the sample was quenching. Consequently, MnS precipitated gradually in the period of mushy zone temperature of current experimental and the in-situ state was obtained. As described before, MnS begins to precipitate when the temperature is lower than the zero ductility temperature (ZDT, *f*_S_ = 0.8). Hence, it is reasonable to say that the growth of MnS inclusions in the present experimental occurred in the low ductility temperature region of 49MnVS steel. The growth time of MnS inclusions for present experimental was shorter than the previous studies and the current simulated results, resulting in smaller size of formed MnS.

Combining the experimental results and calculated values, it can be concluded that the size of MnS inclusions was strongly affected by the cooling rate and rates higher than 100 °C/min were necessary for realizing inclusions which were less than 1.5 μm. With increasing of cooling rate during solidification, the size of MnS grew in the low ductility temperature region (*d*_MnS-H_) and in the whole solidification (*d*_MnS-L_) showed the same trend, and the gap between *d*_MnS-H_ and *d*_MnS-L_ was shrinking. It also shows that rapid cooling condition can preserve the in-situ state of the sample at high temperature. The size of formed MnS increases with decreasing cooling rate, causes the increased difference between *d*_MnS-H_ and *d*_MnS-L_ due to the difference in growth time.

## 5. Conclusions 

A mathematical model has been developed to simulate the solute segregation and growth of manganese sulfide during solidification. Additionally, laboratory experiments were carried out to clarify the effect of cooling rate on the MnS inclusions of resulphurised 49MnVS steel. Based on the simulated results and experimental data, the following conclusions are drawn:

(1) The MnS begins to precipitate in the remnant liquid when local solubility product of manganese and sulfur exceeds the equilibrium value, and the segregation ratio of solute Mn and S are approximately 1.57 and 6.87 at the beginning of MnS formation.
(2) The solute element of S was the determining species in deciding the nucleation and precipitation of MnS, while the solute element Mn affected the precipitated amount of MnS owing to the high partition coefficient in solid phase.
(3) The current coupled model can be used to simulate the growth of MnS inclusions in resulfurized steel, and the calculated size of the formed MnS inclusions fits well with the previous experimental results. The value of the MnS size is evidently affected by cooling rate, and the relationships between *d*_MnS-H_ and *d*_MnS-L_ with *υ* were given by the mathematical expressions as follows: $d_{\text{MnS-H}}=2.040\upsilon^{-0.123}$; $d_{\text{MnS-L}}=7.830\upsilon^{-0.355}$.

## Figures and Tables

**Table 1:** Main chemical composition of steel 49MnVS (wt.%).

C	Si	Mn	P	S	Al	O	N	Cr	Fe
0.48	0.35	0.91	0.013	0.047	0.012	0.0015	0.007	0.2	Balance

**Table 2:** Interaction coefficient between elements in liquid steel.

$e_{i}^{j}$	C	Si	Mn	P	S	Cr	Ni	Mo
Mn	−0.07	0	0	−0.0035	−0.048	-	-	-
S	0.11	0.063	−0.026	0.029	−0.028	−0.011	0	0.0027

**Table 3:** Main kinetic parameters of MnS formation in steels for model calculations.

Parameter	γ-Phase	α-Phase
Lattice parameter of MnS at room temperature (nm)	0.5223	0.2866
Linear expansion efficient of MnS (K^−1^)	1.81 × 10^−5^	1.81 × 10^−5^
Specific interface energy *σ* (J/m^2^)	1.7969 − 0.8097 × 10^−3^ T	0.8157 − 0.2921 × 10^−3^ T
Diffusion activation energy of Mn atom(J)	0.4334 × 10^−18^	0.3653 × 10^−18^
Lattice constant of matrix (nm)	0.3591	0.2863

**Table 4:** Equilibrium partition coefficients and diffusion coefficient of the solute elements.

Element	$k_{\delta /L}$	$k_{\gamma /L}$	$k_{\delta /\gamma }$	*m_i_* (°C pct^−1^)	*n_i_* (°C pct^−1^)	$D_{i}^{\delta }({\mathrm{cm}}^{2}/s)$	$D_{i}^{\gamma }({\mathrm{cm}}^{2}/s)$
C	0.19	0.34	0.56	78	−1122	0.0127exp(−81379RT)	0.0127exp(−81379RT)
Si	0.77	0.52	1.47	7.6	60	8.0exp(−248948RT)	0.3exp(−251458RT)
Mn	0.76	0.78	0.97	4.9	−12	0.76exp(−224400RT)	0.055exp(−249500RT)
P	0.23	0.13	1.75	34.4	140	2.9exp(−230120RT)	0.01exp(−182841RT)
S	0.05	0.035	1.43	38	160	4.56exp(−214600RT)	2.4exp(−224000RT)
